# The Experience in Nicaragua: Childhood Leukemia in Low Income Countries—The Main Cause of Late Diagnosis May Be “Medical Delay”

**DOI:** 10.1155/2012/129707

**Published:** 2012-02-12

**Authors:** C. De Angelis, C. Pacheco, G. Lucchini, M. Arguello, V. Conter, A. Flores, A. Biondi, G. Masera, F. Baez

**Affiliations:** ^1^Pediatric Department, San Gerardo Hospital and University of Milano Bicocca, via Pergolesi 33, 20900 Monza (MB), Italy; ^2^Pediatric Hemato-Oncology Department, Manuel de Jesus Rivera “La Mascota” Hospital, Managua, Nicaragua; ^3^Pediatric Department, “Ospedali Riuniti”, Largo Barozzi 1, 24128 Bergamo (BG), Italy

## Abstract

*Background*. The event-free survival for pediatric leukemia in low-income Countries is much lower than in high-income countries. Late diagnosis, which is regarded as a contributing factor, may be due to “parental” or “medical” delay. *Procedures*. The present study analyses determinants of lag time from first symptoms to diagnosis of leukemia, comparing pediatric (0–16 years old) patients in two referral centers, one in Nicaragua and one in Italy. An observational retrospective study was conducted to assess factors influencing the time to diagnosis. *Results*. 81 charts of children diagnosed with acute myeloid leukemia or lymphoblastic leukemia were analyzed from each centre. Median lag time to diagnosis was higher in Nicaragua than in Italy (29 versus 14 days, *P* < 0.001) and it was mainly due to “physician delay” (16.5 versus 7 days, *P* < 0.001), whereas “patient delay” from symptoms to first medical assessment was similar in the two centers (7 versus 5 days, *P* = 0.27). Moreover, median lag time from symptoms to diagnosis was decreased in Nicaraguan districts were a specific training program upon childhood oncological diseases was carried out (20.5 versus 40 days, *P* = 0.0019). *Conclusions*. Our study shows that delay in diagnosis of childhood leukemia is mainly associated with “physician delay” and it may be overcome by programs of continuous medical education.

## 1. Introduction

Childhood leukemia is the most common childhood cancer, with an annual incidence of 4.5 : 100.000 [1]. The most common leukemia subtype is acute lymphoblastic leukemia (ALL) and the majority of affected patients (80%, 75.000 cases/year) live in low income countries (LIC). There, the estimated 5 years event-free survival (EFS) is only 35%, versus 75% in high income countries (HIC), due to difficulties in diagnosing and treating the disease or inadequate medical facilities and to socioeconomical factors [2–5]. Few studies have analyzed the determinants of lag time in the diagnosis of childhood cancer or leukemia [6–15]. Very few studies have been conducted to assess the impact of “medical delay” on timing of cancer diagnosis and only one research on leukemia in LIC is available to our knowledge [15].

The present study reports the lag time from first symptoms to diagnosis in childhood leukemia in the Referral Center of Nicaragua. The impact of physicians' delay is analyzed and is compared with that of an HIC Center.

## 2. Materials and Methods

The study (observational, retrospective) was run in the context of a twinning program for pediatric hematooncology started in 1986 between La Mascota Hospital in Managua (Nicaragua) and San Gerardo Hospital in Monza (Italy) [16].

Eligible for the study at “La Mascota Hospital” were all children under 16 years of age who were diagnosed with acute myeloid leukemia (AML) or acute lymphoblastic leukemia (ALL) (*n* = 81) between August 2006 and January 2009. Data were obtained from medical records and verified through semistructured interviews with one or both parents. Interviews were conducted by the same physician and took place within 12 months after the diagnosis in the majority (90%) of cases. The pathway from first symptoms to diagnosis was analyzed considering the following items: type of medical structures where the first assessment took place and number of medical evaluations, laboratory or imaging investigations, and treatments performed before the diagnosis. Age, gender, number of family members, family daily income, “parents' education level,” distance from the referral centre (hours of travel), district of origin, and clinical condition at admission were also considered.

A comparison group of Italian children (*n* = 81) diagnosed with ALL or AML between August 2006 and June 2009 at San Gerardo Hospital in Monza was used to assess the impact of “patient” (see below) and “physician” delay in the two countries (Nicaragua and Italy). Data was collected from medical records where information about history from first symptoms to diagnosis are regularly collected interviewing parents at first admission for clinical research purposes.

Selection criteria for this comparison group were the same applied in the study group: 81 consecutive patients who underwent treatment or clinical assessment in the outpatient clinic.

The time interval from first symptoms to diagnosis was defined as “lag time” and classified as follows: “patient delay” and “physician delay.” The former was defined as the time interval from first symptoms to first medical consultation, the latter as the time interval from first consultation to diagnosis [17]. The word “delay” was meant as a time interval, measured in days, without implying whether this interval exceeded a particular threshold of clinical acceptability. Lag time in the population was divided into two time intervals: “0–30 days” and “>30 days” to compare our results with published data.

The type of first medical evaluation was classified into 3 levels: “first” (private doctor, primary healthcare services), “second” (private and public hospitals), and “third” (referral centers: La Mascota Hospital in Managua, Nicaragua and San Gerardo Hospital in Monza, Italy).

Clinical conditions at admission were described as “poor” in presence of mucosal hemorrhagic diathesis, sepsis or need for oxygen therapy; in “good” conditions otherwise.

Nicaraguan patients population was divided into two subgroups using median age as cutoff: “<7 years old” or “≥7 and ≤16 years old”.

Nicaraguan national districts were classified in two categories: “qualified” (districts where a specific training program in pediatric oncology was carried out) and “not qualified”.

### 2.1. Statistical Analysis

Statistical analysis was performed with the EPI INFO software. Correlation between different variables was examined with the chi-square test (*P* significance of 0.05). In case of a small sample (*n* < 5), the Fisher exact test was used. Nonparametric tests (Mann-Whitney/Wilcoxon Two-Sample Test) was used to determine the difference between medians of the independent samples.

## 3. Results

This study included 81 children with leukemia (age 0 to 16 years) from both La Mascota Hospital, Managua (Nicaragua) and San Gerardo Hospital, Monza (Italy). Median age was 7.25 years in the Nicaraguan population and 6.4 years in the Italian one. Males were 41 in Nicaraguan sample and 48 in the Italian one. ALL or AML was diagnosed, respectively, in 76 and 5 Nicaraguan children and in 70 and 11 Italian patients.

Data concerning the time interval from first symptoms to diagnosis and the diagnostic pathway are shown in Tables 1 and 2. Median lag time in Managua was greater than in Monza (29 versus 14 days, *P* < 0.01). “Patient delay” was similar for Nicaraguan and Italian populations (median: 7 versus 5 days, *P* = 0.27), while “physician delay” was significantly greater in Nicaraguan sample (median: 16.5 versus 7 days, *P* < 0.01). Results were similar when ALL and AML subgroups were analyzed separately (Table 1).

**Figure 1:**
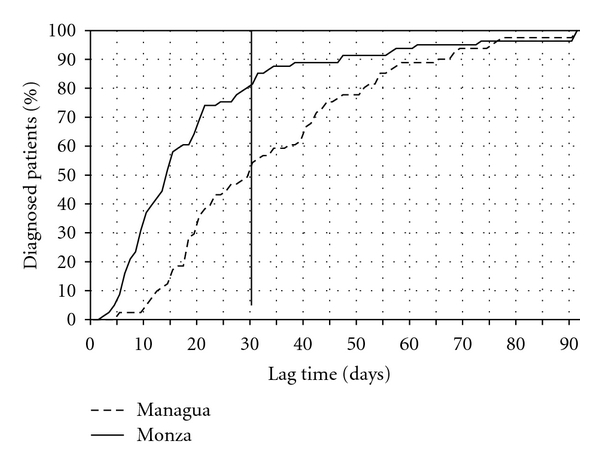
Lag time to diagnosis in days: cumulative percentage.

Overall the percentage of cases diagnosed >30 days after the onset of symptoms was significantly higher in Managua (45.7% versus 18.5%, *P* < 0.01) (Table 1 and Figure 1).

Nicaraguan patients who came from “qualified districts” were diagnosed within a shorter lag time than those from the remaining districts (median lag time: 20,5 versus 40 days, *P* < 0.01 Mann-Whitney/Wilcoxon test).

There was a higher percentage of Nicaraguan children with poor condition at admission compared to Italian ones (*P* = 0.013). In both populations the most frequent first medical aid was given at a primary healthcare facility. However Nicaraguan patients were checked more times (*P* < 0.01) and more frequently received medical treatment such as antibiotic therapies (*P* = 0.04) and iron supplementation (*P* < 0.01) before diagnosing leukemia (Table 2).

Age was associated with lag time: younger patients (<7 years old) had higher probabilities of being diagnosed at least 30 days after the onset of first symptoms compared to the older ones (57% versus 33%, *P* = 0.03, Chi-square test).

## 4. Discussion

Leukemia is one of the leading causes of disease-related death in children living in HIC, but it represents an important public health concern also in LIC, where difficulties in diagnosing and treating childhood malignancies undermine the outcome (5 years EFS 75% versus 35% in HIC at LIC, respectively, [2–4]). Delay in diagnosis could be a contributing factor in this context.

In our study we found that median lag time from the first symptoms to diagnosis of childhood leukemia in La Mascota Hospital, Managua—Nicaragua was significantly higher than in San Gerardo Hospital, Monza, Italy (29 versus 14 days, *P* < 0,  001) with a significantly higher percentage of Nicaraguan patients diagnosed after >30 days compared to Italian ones (45,7% versus 18,5%) (Figure 1). This delay may be a contributing factor to the worse clinical conditions at admission of Nicaraguan children compared to Italian ones (*P* = 0.013), although the worse health status of childhood population in Nicaragua has also to be taken into account [18].

These results are consistent with data already reported in the literature [8] (Table 3), which describe a median lag time to diagnosis for leukemia of 2 weeks in HIC and 4 weeks in LIC [17].

We therefore analyzed lag time composition: median “patient delay” (5 versus 7 days) does not differ in the two countries and is comparable to data available for HIC (Table 3); “physician delay” is on the other hand higher in Nicaragua compared to Italy. Data collected in Nicaragua are similar to the results reported by Stefan et al. from an urban area of South Africa [15].

“Physician lag time” for Nicaraguan patients was higher despite the higher frequency of medical examinations and prescriptions.

This may be due to a different medical expertise towards oncologic diseases in childhood and to confounding symptoms of common infectious diseases [19–22].

 Of importance, the socioeconomic parameters, which are known to play an important role on prognosis in Nicaragua [23–26], did not influence lag time to diagnosis in our study. The present study also shows that Nicaraguan children <7 years old were diagnosed with a greater lag time than the older ones (up to 16 years of age). These data are interesting because, in presence of a significant correlation with age, the opposite has been reported for childhood solid tumors both in LIC and in HIC [6, 8–11]. This could be due to older patients' ability to better describe specific symptoms or to the lack of regular medical care for younger children.

Other authors found a correlation between distance from referral centre and lag time, both in HIC and in LIC [8, 10]. In our study the distance from Managua did not influence the time to diagnosis, but there was a significant difference in lag time according to the district of origin. Nicaraguan patients from “qualified districts” were diagnosed earlier than those from the remaining districts. This data underlines the importance of specific training programs in childhood cancer for physicians working in first or second level health care centers.

Unfortunately we do not have yet data about correlation between “lag time to diagnosis” and Event Free Survival (EFS) rate in Nicaragua; for this reason the potential impact of our findings on outcome has still to be determined and should be investigated in the future.

## 5. Conclusions

In conclusion, this study, although based on relatively small samples, shows that lag time from first symptoms to childhood leukemia diagnosis is significantly greater in patients admitted at “La Mascota Hospital” (Managua, Nicaragua) compared to those admitted at “San Gerardo Hospital” (Monza, Italy); the lag time is mostly due to “physician delay.” Diagnosis delay in LIC may be influenced by the fact that symptoms of leukemia at the onset might resemble other diseases which have a high prevalence in these countries (i.e., infectious diseases, anemia, and malnutrition). Lag time in Nicaragua seems to be associated to a low suspicion of the oncologic disease that may be improved by specific programs for medical training in first level health care centers. Thus the present study suggests that specific educational programs for physicians may be the most important mean to decrease the diagnostic lag time for childhood leukemia in LIC.

## Figures and Tables

**Table 1:** Time intervals from first symptoms to diagnosis of leukemia.

	Managua	Monza	*P* value
Lag time from first symptoms to diagnosis (days)			
Median, (IQR)	29 (18–44)	14 (9–24)	^#^ *P* < 0.01
ALL	29,5 (18–45)	15 (9–27)	^#^ *P* < 0.01
AML	21 (18–39)	9 (6–20)	^#^ *P* = 0.03

Patient delay (days)			
Median, (IQR)	7 (1,5–15)	5 (1–13)	^#^ *P* = 0.27
ALL	7 (1,5–15)	5,5 (1–13)	^#^ *P* = 0.5
AML	6 (3–11)	4 (2–13)	^#^ *P* = 0.9

Physician delay (days)			
Median, (IQR)	16.5 (7–33,5)	7 (2–4)	^#^ *P* < 0.01
ALL	16 (7–33)	8 (2–15)	^#^ *P* < 0.01
AML	18 (6–33)	3 (1–6)	^#^ *P* = 0.03

Lag time to diagnosis: “0–30 days” and “>30 days” groups			
Lag time to diagnosis “>30 days” (frequencies)	37 (45.7%)	15 (18.5%)	°*P* < 0.01
Lag time to diagnosis “0–30 days” (frequencies)	44 (54.3%)	66 (81.5%)	

°**:** Chi-square test; ^#^ **:** Mann-Whitney/Wilcoxon Two-Sample Test; ALL**:** acute lymphoblastic leukemia; AML**:** acute myeloid leukemia.

**Table 2:** Medical assessments performed before diagnosing leukemia and clinical condition at the diagnosis.

	Managua	Monza	*P* value
Number of medical evaluations before diagnosis			
Median, (IQR)	3 (2–4)	2 (2-3)	^#^ *P* < 0.01

Clinical conditions at admission (frequencies)			
Good	52 (64.2%)	71 (87.7%)	°*P* = 0.013
Poor	29 (35.8%)	10 (12.3%)	

Type of first medical evaluation before diagnosis (frequencies)			
First	51 (63%)	57 (70.4%)	°*P* = 0.32
Second	29 (35.8%)	23 (28.4%)	°*P* = 0.31
Third	1 (1.2%)	1 (1.2%)	^•^ *P* = 0.75

Antibiotics treatment before diagnosis (frequencies)			
Yes	48 (59%)	35 (43%)	°*P* = 0.04
No	33 (41%)	46 (57%)	

Iron supplementation before diagnosis (frequencies)			
Yes	16 (20%)	0 (0%)	^•^ *P* < 0.01
No	65 (80%)	81 (100%)	

°**:** Chi-square test; ^•^ **:** Fisher Test; ^#^ **:** Mann-Whitney/Wilcoxon Two-Sample Test.

**Table 3:** Literature notes about studies dealing with lag time from first symptoms to diagnosis in childhood leukemia.

	Study	Diagnostic delay, median (IQR), days			Population diagnosed after 30 days, %	Characteristic of population
		Patient delay	Physician delay	Lag time		
HIC (countries with GNP per capita income of $12.196 or more according to the World Bank Classification)	Klein Geltink et al. [10] Canada	7 (1–19)	1 (0–11)	NA*	NA*	826 patients aged 0–15 (1995–2000)
	Thulesius et al. [12] Sweden	7 (0–91)	0 (0–63)	21 (0–105)	24%	25 patients aged 0–16 (1984–1995)
	Saha et al. [11] United Kingdom	NA*	NA*	21 (7–224)	NA*	65 patients aged 0–15 (1982–1990)
	Flores et al. [9] U.S.A.	NA*	NA*	NA*	18,7%	123 patients aged 0-20 (1976–1984)
	Dang-Tan et al. [6] Canada	8 (1–21)	3 (1–14)	19 (9–36)	NA*	944 patients aged 0–19 (1995–2000)
	De Angelis et al. (this paper) Italy	5 (1–13)	7 (2–4)	16.5 (7–33,5)	18,5%	81 patients aged 0–16 (2006–2009)

LIC	Fajardo-Gutiérrez et al. [8] Mexico	NA*	NA*	NA*	47%	1676 patients aged 0–14 (1981–1992)
	B. O. James et al. [14] Nigeria	NA*	NA*	56 (14–252)	NA*	9 patients aged 1–14 (2006–2008)
	Stefan et al. [15] South Africa	4 (NA*)	22 (NA*)	31 (NA*)	NA*	63 patients aged 0–15 (2000–2009)
	De Angelis et al. (this paper) Nicaragua	7 (1,5–15)	16.5 (7–33,5)	29 (18–44)	45,7%	81 patients aged 0–16 (2006–2009)

*NA: not available.
